# Developing, implementing, and monitoring tailored strategies for integrated knowledge translation in five sub-Saharan African countries

**DOI:** 10.1186/s12961-023-01038-x

**Published:** 2023-09-04

**Authors:** Kerstin Sell, Nasreen S. Jessani, Firaol Mesfin, Eva A. Rehfuess, Anke Rohwer, Peter Delobelle, Bonny E. Balugaba, Bey-Marrié Schmidt, Kiya Kedir, Talitha Mpando, Jean Berchmans Niyibizi, Jimmy Osuret, Esther Bayiga-Zziwa, Tamara Kredo, Nyanyiwe Masingi Mbeye, Lisa M. Pfadenhauer

**Affiliations:** 1 Institute for Medical Information Processing, Biometry and Epidemiology, LMU Munich, Elisabeth-Winterhalter-Weg 6, 81377 Munich, Germany; 2 Pettenkofer School of Public Health, Munich, Germany; 3 Centre for Evidence-Based Healthcare, Division of Epidemiology and Biostatistics, Faculty of Medicine and Health Sciences, Stellenbosch University, Cape Town, South Africa; 4 Department of International Health, Johns Hopkins Bloomberg School of Public Health, Baltimore, MD USA; 5 Non-Communicable Diseases Directorate, Armauer Hansen Research Institute, Addis Ababa, Ethiopia; 6 Chronic Diseases Initiative for Africa, University of Cape Town, Cape Town, South Africa; 7 Department of Public Health, Vrije Universiteit Brussel, Brussels, Belgium; 8 Department of Disease Control and Environmental Health, School of Public Health, College of Health Sciences, Makerere University, Kampala, Uganda; 9 School of Public Health, University of the Western Cape, Cape Town, South Africa; 10 Health Systems Research Unit, South African Medical Research Council, Cape Town, South Africa; 11 School of Global and Public Health, Kamuzu University of Health Sciences, Blantyre, Malawi; 12 College of Medicine and Health Sciences, University of Rwanda, Kigali, Rwanda; 13 Department of Global Public Health, Karolinska Institutet, Stockholm, Sweden; 14 Division of Epidemiology and Biostatistics and Division of Clinical Pharmacology, Faculty of Medicine and Health Sciences, Stellenbosch University, Cape Town, South Africa

**Keywords:** Public health, Integrated knowledge translation, Implementation, Evaluation, Non-communicable diseases, Uganda, South Africa, Malawi, Ethiopia, Rwanda

## Abstract

**Background:**

Integrated knowledge translation (IKT) through strategic, continuous engagement with decision-makers represents an approach to bridge research, policy and practice. The Collaboration for Evidence-based Healthcare and Public Health in Africa (CEBHA +), comprising research institutions in Ethiopia, Malawi, Rwanda, South Africa, Uganda and Germany, developed and implemented tailored IKT strategies as part of its multifaceted research on prevention and care of non-communicable diseases and road traffic injuries. The objective of this article is to describe the CEBHA + IKT approach and report on the development, implementation and monitoring of site-specific IKT strategies.

**Methods:**

We draw on findings derived from the mixed method IKT evaluation (conducted in 2020–2021), and undertook document analyses and a reflective survey among IKT implementers. Quantitative data were analysed descriptively and qualitative data were analysed using content analysis. The authors used the TIDieR checklist to report results in a structured manner.

**Results:**

Preliminary IKT evaluation data (33 interviews with researchers and stakeholders from policy and practice, and 31 survey responses), 49 documents, and eight responses to the reflective survey informed this article. In each of the five African CEBHA + countries, a site-specific IKT strategy guided IKT implementation, tailored to the respective national context, engagement aims, research tasks, and individuals involved. IKT implementers undertook a variety of IKT activities at varying levels of engagement that targeted a broad range of decision-makers and other stakeholders, particularly during project planning, data interpretation, and output dissemination. Throughout the project, the IKT teams continued to tailor IKT strategies informally and modified the IKT approach by responding to ad hoc engagements and involving non-governmental organisations, universities, and communities. Challenges to using systematic, formalised IKT strategies arose in particular with respect to the demand on time and resources, leading to the modification of monitoring processes.

**Conclusion:**

Tailoring of the CEBHA + IKT approach led to the inclusion of some atypical IKT partners and to greater responsiveness to unexpected opportunities for decision-maker engagement. Benefits of using systematic IKT strategies included clarity on engagement aims, balancing of existing and new strategic partnerships, and an enhanced understanding of research context, including site-specific structures for evidence-informed decision-making.

**Supplementary Information:**

The online version contains supplementary material available at 10.1186/s12961-023-01038-x.

## Background

Integrated knowledge translation (IKT) constitutes an approach for researchers to work with knowledge users and has been proposed as one of the approaches to facilitate evidence-informed decision-making (EIDM) in healthcare [1, 2]. Described as an “ongoing relationship between researchers and decision-makers (clinicians, managers, policy-makers, etc.) for the purpose of engaging in a mutually beneficial research project […] to support decision-making” [3], IKT is intended to lead to the generation of evidence that is relevant and useful for decision-making, whilst promoting the use of contextualised research by a receptive audience of decision-makers in the policy-and-practice community [4]. In contrast to other approaches to collaborative research that involve a broad range of stakeholders including citizens and patients [5], knowledge users involved in IKT are described as those “who identify a problem and have the authority to implement the research recommendations” [1].

IKT practice and the accompanying research have a lot of traction, including interest from both funders [6–8], who want to address the under-utilisation of research findings and improve the uptake of research evidence [9], and policy-makers, who are tasked with increasing the accountability for public spending on research [10] and, ultimately, with enhancing health system performance and population health [3].

Originally coined and developed in the Canadian health research context [5], IKT advances the concept of knowledge translation (KT), which has been defined as the “synthesis, exchange, and application of knowledge by relevant stakeholders to accelerate the benefits of global and local innovation in strengthening health systems and improving people’s health.” [11]. Whilst KT in practice may be dissemination-centred, IKT emphasises continuous stakeholder engagement [12]. In IKT, knowledge users may thus partake in formulating the research question, help shape the methodology and tools, contribute to data collection and interpretation, and support results dissemination [13]. Of note, such research co-production endeavours do not come without their own barriers and challenges, including but not limited to often substantial time and resource requirements, tokenistic engagement, and conflicts related to roles and power [14–16]. With its focus on continuous interaction with decision-makers, IKT shares some characteristics with other approaches such as knowledge brokers, KT networks and knowledge [translation] platforms [8, 17–22]. Whilst IKT has been incorporated in diverse research endeavours in the past decade [3, 10] it remains an emerging field of research inquiry. Evidence for building IKT theory, informing IKT methods including approaches to designing and evaluating an IKT intervention, optimising the IKT process, and building meaningful and equal partnerships is still needed [23].

From 2017 to 2022, the German Federal Ministry of Education and Research supported the Collaboration for Evidence-Based Healthcare and Public Health in Africa (CEBHA +) to conduct policy- and practice-relevant research. CEBHA + is a research consortium with nine partner institutions in Ethiopia, Germany, Malawi, Rwanda, South Africa, and Uganda [24] and focuses on non-communicable diseases (NCDs), including the prevention, diagnosis and integrated care of cardiovascular disease, diabetes, hypertension, and road traffic injuries. An overview of CEBHA + research tasks is available in Additional file 1.

CEBHA + aims to promote EIDM in the African partner countries by (i) identifying relevant and context-sensitive research priorities; (ii) conducting robust, internationally competitive research; and (iii) linking primary research with evidence synthesis, implementation research, and policy and practice [25]. CEBHA + hence positioned itself to produce both ‘global’ and ‘local’ evidence [26] and aspired to act as a trusted entity to provide decision-makers with high- quality, timely, and contextualised evidence [27]. To work towards these goals, CEBHA + partners started engaging key stakeholders^1^ in defining research priorities and research questions for the CEBHA + body of work at the proposal writing stage [25].

The authors adopted an IKT approach to facilitate collaboration with decision-makers throughout the project. Following the development of a generic IKT approach for the network (item 2, see below), and training in EIDM and IKT [28, 29] country teams developed and tailored five site-specific IKT strategies. Some experiences with the implementation and outcomes of these strategies have been published elsewhere [30–32].

When CEBHA + set out on planning IKT in 2017, there was a dearth of descriptions of how to *do* IKT, both in peer-reviewed and grey literature [3]. The objective of this paper is therefore to provide a structured account of the CEBHA + IKT approach: the development, implementation, monitoring of site-specific IKT strategies, and subsequent reflection of the approach across CEBHA + sites.

## Methods

The study is guided by a conceptualisation of IKT as a complex intervention in a complex system [33]. The IKT approach does not, however, constitute a single intervention, but rather entails a multi-pronged ensemble of tailored interventions, as detailed in the five site-specific IKT strategies. The implementation of the CEBHA + IKT approach is complemented by a process and outcome evaluation, undertaken by project partners not involved in IKT implementation in the African countries (KS, ER, LMP). This evaluation is intended to shed light on whether or not such an extensive approach to IKT leads to increased uptake and relevance of scientific evidence and on how this approach worked [34]. In the present article, we use the TIDieR checklist [35] to complement this evaluation by examining and reporting how the overall IKT approach and site-specific IKT strategies were developed, adapted, implemented and monitored in a systematic and structured way. The TIDieR checklist comprises twelve items (see below) and was developed to report on interventions in a transparent manner to facilitate their replication, adaptation, or scale [35].

### Data collection

We draw on three primary data sources (below and in Tables 1 and 2) to report on these twelve items. Of note, not all authors were involved in all areas of data collection as they were respondents for the surveys and participants in the evaluation. In order to avoid any conflicts of interest, those authors responsible for IKT at their institutions were invited to contribute to the analyses of anonymised and aggregated data and the writing of the paper. We therefore provide authors’ initials for each of the data collection and analysis steps described below.

- **Mixed methods evaluation**: In the early-stage IKT evaluation, KS and LMP collected responses from 31 surveys and 33 interviews from CEBHA + researchers and their IKT partners from policy and practice [34]. For this publication, we drew on evaluation data corresponding to TIDieR framework items, i.e. codes covering frequencies and mode of interaction, implementation process, and the intervention context, specifically macro-level context like EIDM structures.
- **IKT documents:** Forty nine IKT-related documents were available for document analysis conducted by KS and LMP. These included: the minutes of quarterly IKT team meetings, as well as ‘IKT updates’, site-specific IKT documents with details on stakeholder engagement, IKT activities, and reflections about IKT and stakeholder engagement. Additionally, Microsoft Excel-based IKT strategies, and further documentation of IKT activities from each of the African CEBHA + sites were collected.
- **Reflective survey**: LMP and KS created an online reflective survey for the IKT team which covered items from the TIDieR checklist that were not sufficiently covered by other data sources (see Additional file 2). The survey comprised both open-ended and closed questions with multiple answer options. The survey was sent to all CEBHA + IKT focal points (NSJ, AR, BEB, PD, KK, TM, JBN, SN), responsible for coordinating IKT activities at implementation sites. This yielded 8 responses. IKT focal points who are authors on this paper were not privy to the identifiable surveys.

**Table 1:** Overview of data sources and responses

Data sources (timing of data collection)	Respondents/number of documents
Mixed methods evaluation data: Semi-structured interviews and online survey (March 2020–January 2021)	• Semi-structured interviews (*n* = 33) ⚬ CEBHA + researchers: *n* = 25 ⚬ Stakeholders: *n* = 7 • Online Survey (*n* = 31) ⚬ CEBHA+ Researchers: *n* = 24 ⚬ Stakeholders: *n* = 7
IKT documents (March 2020–November 2022)	Documents capturing IKT activities (*n* = 49) • IKT quarterly update documents: *n* = 25 • IKT meeting and other minutes: *n* = 11 • Microsoft Excel-based IKT strategies: *n* = 13
IKT focal point reflective survey (September–November 2022)	Responses to survey (*n* = 8) • Ethiopia: *n* = 1 • Malawi: *n* = 1 • Rwanda: *n* = 2 • South Africa: *n* = 3 • Uganda: *n* = 1

In addition to these data sources, we drew on CEBHA + IKT experiences and previously published research [30–32]

**Table 2:** Summary of TIDieR items and data sources

Item no	Data sources			Summary
	Mixed methods evaluation	Document analyses	Reflective survey	
1: Name	–	–	–	CEBHA + IKT approach & site-specific IKT strategies
2: Why? Rationale, theory and goals	–	–	–	CEBHA + aims to produce contextually relevant research and to enhance the use and uptake of high quality evidence into public health decision-making through continuous engagement with stakeholders. A programme theory was developed for this approach
3 & 4: What pro- cesses, activities and materials were used?	–	●	●	Intervention planning included the development of the CEBHA + IKT approach and its subsequent operationalisation through the development of five site-specific IKT strategies. Implementation of IKT strategies included interacting with decision-makers throughout the research project on a range of occasions. Training material included guidance for researchers on stakeholder engagement and IKT, templates for stakeholder analysis and IKT strategies. Throughout the project period, reports and presentations, publications, issue and policy briefs were materials used to communicate with stakeholders about project progress and results
5: Who undertook IKT?	–	–	●	All CEBHA + researchers, PIs and Co-PIs participated in IKT activities. IKT expertise across the consortium was initially scant but was subsequently built through training and forming a community of practice on IKT
6: How? (mode of delivery)	●	●	●	IKT activities included face-to-face interactions including workshops, briefings, meetings, conferences; phone, email and social media interactions as well as virtual meetings and webinars
7: Where was IKT implemented? (context)	●	●	●	Most IKT activities took place in the capital and major cities of the African CEBHA + countries. Infrastructure both at the level of CEBHA + institutions as in the broader local context influenced IKT activities. Researchers were able to draw on many pre-existing contacts as well as formal and informal connections between decision-makers and their institutions. The COVID-19 pandemic disrupted planned IKT activities and led to other, typically virtual, engagements, related to the pandemic response
8: When and how much?	–	–	–	With the CEBHA + IKT approach launched in 2019, IKT activities were initiated at different time points (2017–2019), often before a formal IKT strategy was finalised. The frequency of IKT activities depended on stakeholder preferences and planning as detailed in the IKT strategies as well as contextual circumstances for more ad hoc interactions
9: Tailoring of IKT strategies	●	●	●	All IKT strategies underwent tailoring that was (i) stakeholder-related, (ii) project-related, or (iii) related to macro-level changes, in particular with respect to the COVID-19 pandemic
10: Modifications of the IKT approach	●	●	●	The IKT approach underwent modification over the course of the project, with the three main modifications being (i) ad hoc engagements complementing planned engagements, (ii) an extension of the understanding of IKT partners in CEBHA + to non-decision-makers, and (iii) a modification of the monitoring processes to be more feasible
11: How well was the IKT approach implemented? (planned)	–	–	–	Fidelity and adherence to the local IKT strategies was planned to be captured during monitoring and IKT strategy updates. Fidelity and adherence to the overall, systematic IKT approach was examined in the IKT evaluation
12: How well was the IKT approach implemented? (actual)	●	●	●	Continuous stakeholder engagement was implemented at all five CEBHA + sites, in particular at the early project phases of defining the research question and methods and requesting research approval from relevant authorities as well as during data interpretation and dissemination. Actual fidelity to the plans varied across sites. Adaptation to changing circumstances allowed for a stronger tailored approach. For instance, many ad hoc engagements occurred linked to the COVID-19 pandemic context. Fidelity to formal monitoring aspects of the IKT approach was less strong

### Data analysis

We undertook a cross-case comparison with a within-site analysis followed by a between-site analysis [36, 37], with each of the five sites—i.e. Ethiopia, Malawi, Rwanda, South Africa (comprising three CEBHA + institutions), and Uganda—representing a case. Initially, insights were analysed separately according to the data source (reflective survey, documents, mixed methods evaluation), data type (quantitative, qualitative), and site.

For the analysis of mixed methods evaluation data and documents we followed the procedures outlined in our evaluation protocol [34]: we employed qualitative content analysis [38] and descriptive quantitative methods to analyse the data derived from semi-structured interviews, surveys and documents. Qualitative data were transcribed, pseudonymised and imported into ATLAS.ti [39]. After familiarisation with the data, KS deductively applied an a priori coding frame based on programme theory constructs and categories [34]. KS developed further codes and categories inductively, thereby expanding the coding frame. This coding frame was then applied and tested by evaluation co-researchers (JO, EB) leading to further expansion and refinement. KS and LMP applied this coding frame in a final coding round of all documents and interviews. Subsequently, they examined the respective codes individually per site and across sites. KS and LMP analysed frequencies of survey responses descriptively. They provided a narrative summary of evaluation items relevant to this publication.

For the analysis of the reflective survey, FM, KS and LMP compiled and organised the data into a table that was structured by TIDieR items and disaggregated by CEBHA + site (i.e. cases). Closed survey questions were analysed descriptively, providing simple frequencies of responses that were subsequently summarised narratively. Open survey questions were analysed following abridged procedures for qualitative content analysis, identifying key themes per site and subsequently comparing themes across sites as part of the between-site analysis. Common features and differences related to identified themes were then summarised in either narrative or tabular format.

Based on data derived from the three data sources and respective analyses, the authors reflected on and discussed the features making up individual TIDieR items. This iterative process resulted in the final description of the IKT approach and IKT strategies.

All qualitative data were analysed in the software ATLAS.ti Windows (Version 9.1.7.0) [39]. Quantitative data were analysed descriptively in Microsoft Excel.

## Results

Below we describe the IKT approach and strategies according to TIDieR items. A summary of all TIDieR items and underlying data sources is available in Table 2.

### Item 1: Brief name

CEBHA + IKT approach & site-specific IKT strategies.

### Item 2: Why a CEBHA + IKT approach? Rationale, theory and goals

This section describes the rationale and theory underpinning the overall CEBHA + IKT approach and site-specific IKT strategies.

#### Rationale and goal

From its conception, indeed from setting research priorities for the consortium with key stakeholders [25], the CEBHA + team were committed to engaging with stakeholders to (a) ensure the relevance of their research and (b) enhance the use and uptake of high quality, contextualised evidence into public health and healthcare decision-making. Originally conceptualised as a research co-production approach, team discussion at the project onset led to identifying IKT as the most suitable approach for stakeholder engagement. Undertaking IKT was then defined as a deliverable, for example, by setting policy dialogues and policy briefs as required research outputs. One research task was dedicated to the implementation and evaluation of IKT (Additional file 1).

#### Theory

**Fig. 1:**
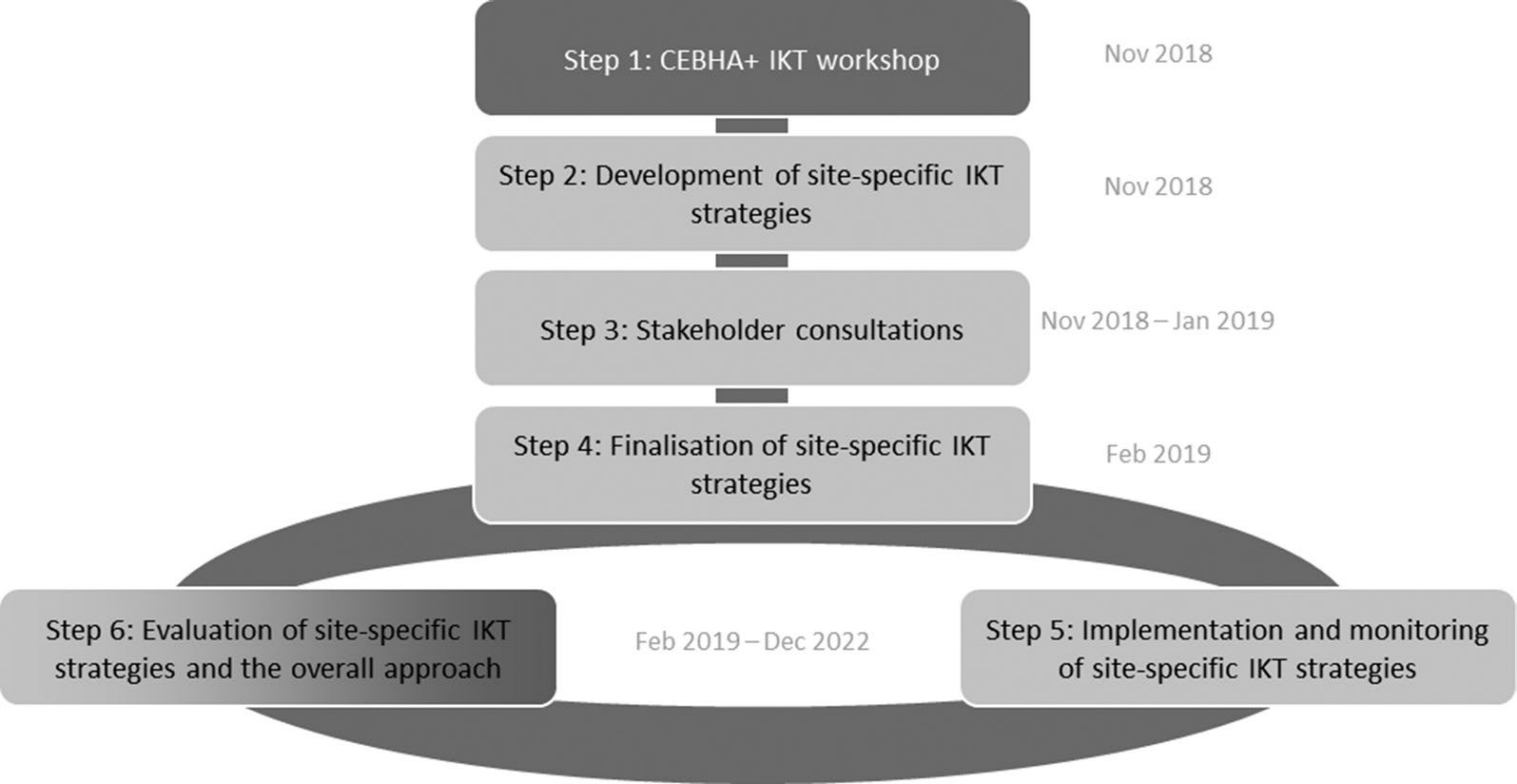
CEBHA + IKT approach [34]

A generic IKT approach was developed to facilitate a coordinated approach and implementation across the research consortium (Fig. 1, [34]) to provide a canvas for site-specific tailoring later. A scoping review by Gagliardi and colleagues provided an overview of evaluations of IKT approaches, relevant enablers and barriers, preconditions, and outcomes [3] and served as a starting point to develop the programme theory. Additional IKT and implementation science literature informed the advancement of this theory [33, 40].

This CEBHA + IKT programme theory captured the intended intervention outcomes, implementation outcomes, intermediate outcomes as well as final outcomes: Intervention outcomes included relationship enhancement (improved mutual understanding and trust, changed attitudes towards research/policy-and-practice), collaborative research (appreciation, diversity of partners, continuous engagement), and capacity-building (improved access to information and contacts, broadened perspective and skills, enhanced capacity for collaboration) [34]. We theorised that these would lead to long-term and continuous partnerships between researchers and decision-makers. Ultimately, these would result in the production of research that was more relevant for decision-makers and implemented more rapidly in policy and practice [3, 33]. The theory also captured contextual factors on the individual, organisational, project, and macro level. As such, it constituted an initial shared understanding of how the CEBHA + IKT approach was envisioned to work as well as making its goals explicit.

This programme theory informed a coordinated, multistep approach to developing, implementing and evaluating our IKT approach. Recognising the broad range of existing relationships, diverse IKT and KT activities at the different institutions and the breadth of CEBHA + research tasks, the consortium did not design a uniform IKT intervention but an approach that was then tailored to each CEBHA + site.

### Item 3 and 4: What? Processes, activities, and materials used in the IKT approach

#### Development of IKT strategies

The CEBHA + IKT approach and development of the five tailored, site-specific IKT strategies involved five steps (Fig. 1).

All CEBHA + researchers were given the opportunity to participate in an IKT workshop (**Step 1**, Fig. 1) in the early project phase. This workshop was adapted from an existing short course—*“Evidence-Informed Decision-Making: The Art, Science and Complexity of (Integrated) Knowledge Translation”—*offered at Stellenbosch University (SU) [28, 29]. In addition to introducing participants to KT resources available in the public domain (e.g. [41–43], it focused on undertaking a stakeholder analysis and using a stakeholder engagement matrix for developing the CEBHA + IKT strategies. A concept paper for the CEBHA + IKT approach including guidance for developing the site-specific IKT strategies was made available to participants [44].

The workshop was attended by seventeen CEBHA + researchers from all sites and kicked off the development process of the site-specific IKT strategies (**Steps 2–4**). These resulted from a structured process of brainstorming among the five teams to identify relevant stakeholders for the CEBHA + research tasks at their implementing site. Subsequently, the interest and power of these individuals were analysed [45] and stakeholders were prioritised. For prioritised stakeholders, the stakeholder engagement plan (= IKT strategy) was developed, constituting an initial plan of how these individuals should be approached, detailing the aim of the engagement, the message, forum, timing, messenger, and resources needed [28]. Consideration of the research team’s IKT capacity and pre-existing contacts informed this plan. Consultations with these stakeholders served as a feedback mechanism to clarify interest and preferences for engagements and finalise the initial IKT strategies, and enabled identification of further relevant stakeholders.

CEBHA + researchers sought to involve decision-makers and other stakeholders early on and throughout the research process to allow for continuous involvement (**Step 5**). Monitoring of these IKT activities was planned to help identify successful interactions and inform IKT strategy updates. However, this was implemented differently than intended, as we describe under modifications (item 10). The evaluation (**Step 6**) was designed to inform the refinement of the IKT approach and strategies. Through monitoring and evaluation efforts (**Steps 5 and 6**), IKT strategies and implementation were theorised to be adapted iteratively as the project progressed.

#### Site-specific IKT strategies

The five site-specific IKT strategies developed in this process were heterogeneous as the prioritised stakeholders, rationale and key characteristics were tailored to local context (Table 3).

**Table 3:** Overview of priority stakeholders, key characteristics and rationale of IKT strategies

**Ethiopia**: The Ethiopian IKT strategy focused on strengthening and establishing partnerships with policy-makers at the Ethiopian Ministry of Health (MoH), non-governmental organisations (NGOs) active in NCD healthcare delivery, advocacy organisations, and other academic institutions in order to: (a) share project and research updates with those active in the NCD space, (b) collaborate on capacity-building activities for strengthening EIDM, and (c) catalyse the establishment of a national NCD research network that integrates the efforts of universities, the MoH, and other stakeholders to prevent and control NCDs, as well as to bridge the evidence-policy-practice gap
**Malawi**: The MoH’s NCD department sets the national NCD research agenda and the ministry’s knowledge translation platform (KTP) facilitates a dialogue between policy-makers, researchers and frontline clinicians for EIDM. These two departments were therefore defined as priority stakeholders to: (a) strengthen the existing relationship with policy-makers in those two MoH departments and, (b) facilitate the implementation of the CEBHA + activities. The CEBHA + in Malawi collaborated with the KTP on evidence synthesis, capacity-building, and dissemination of research results
**Rwanda**: The site-specific IKT strategy prioritised engagement with the MoH, Rwanda Biomedical Center (the implementing agency of the MoH), Rwanda Utility Regulatory Authority, Rwanda Transport Development Agency, the Ministry of Public Service and Labor, the Ministry of Sports, and the Traffic Police Department. The rationale for this prioritisation was to (a) facilitate the project implementation process and (b) to collaborate on research activities. IKT activities hence included convening of stakeholder meetings to introduce the CEBHA + project, to report on research activities, to share (preliminary) results, and to receive feedback; co-facilitating data collectors’ trainings; as well as collaborating on manuscript write-up, capacity-building, issue brief development, and dissemination
**South Africa**: The main aim of the South African IKT strategy was to increase the uptake of research evidence in policy and practice. Although the team identified many stakeholders, priority stakeholders were NCD directors in the national and provincial departments of health responsible for developing NCD policies. CEBHA + staff at all three South African institutions had existing relationships with these decision-makers. Activities included stakeholder meetings at various points of the project, an NCD symposium to bring together all stakeholders in 2020, policy dialogues in 2022, conference presentations, peer-reviewed publications, sharing of research results through webinars, issue briefs, emails and in-person meetings
**Uganda**: The Ugandan IKT strategy built on the team’s long-standing engagements and experience in road safety work. With the introduction of the IKT approach, the team formalised the engagements with stakeholders and meetings with key stakeholders were subsequently documented which had not been done earlier. With IKT, stakeholder engagements moved away from being more individualistic to institution-based to address staff turnover. Initial communications with stakeholders happened through formal letters which were followed up with phone calls or physical visits. In-person meetings were convened for planning or dissemination

#### Support activities

Further IKT support activities were set up. This activity included online meetings of the IKT team (see item 5) to discuss the current status of IKT, questions, challenges, and successes of IKT (every three months); site-specific IKT team meetings; IKT meetings during the annual project meeting; opportunities for CEBHA + researchers to participate in KT courses, including an introduction to IKT and writing issue briefs [28, 46]; and on-demand support from IKT team leads. Other opportunities for IKT support and learning were presented, for example, in the IKT symposium at the Cochrane Indaba in Cape Town in March 2019, which several CEBHA + staff attended.

#### Materials used in the IKT activities

As part of IKT activities, a range of planning as well as communication materials were used, including the CEBHA + newsletter, study protocols and scientific publications, study reports, workshops, presentations, issue briefs and evidence/policy briefs [47], as well as blogs.

### Item 5: Who undertook IKT?

The CEBHA + IKT approach was implemented by an initial group of seven universities and academic institutions (Table 5). In Malawi, the Epidemiology and Intervention Research Unit (MEIRU) became involved through their partnership with Kamuzu University of Health Sciences (KUHeS), hence expanding the African CEBHA + network to eight institutions.

The CEBHA + IKT team consisted of KT experts based at SU and at LMU Munich and IKT focal points at the other African institutions. The KT experts developed the IKT approach, provided training and support, and facilitated IKT team meetings. IKT focal points were responsible for developing the blueprint for site-specific IKT strategies with their respective local teams and driving the implementation, adaptation and monitoring of IKT strategies. They also shared site-specific IKT updates, challenges and successes with the broader IKT team. The KT group based at LMU Munich was further responsible for conducting an evaluation of the CEBHA + IKT approach [34].

All CEBHA + researchers were involved in engagements with decision-makers. The Ethiopian team also involved individuals who were not part of the CEBHA + project as intermediaries to interact with decision-makers. Whilst formal IKT and KT expertise across the network was scant at the onset, IKT focal points at the end of the project described their IKT experience as moderate to expert and the IKT experience of their wider teams as moderate.

### Item 6: How? Modes of delivery of IKT activities

There were diverse modes of delivering IKT activities. All sites used face-to-face meetings to interact with stakeholders, as well as emails, phone calls or messaging (Table 4). Often, an initial in-person introductory meeting with stakeholders was conducted. Subsequent interactions included updates on the research process and needs-based consultations at different project stages (item 8). Further modes of delivery included interacting through virtual meetings (necessitated by COVID-19 or geographic distance), webinars, twitter and other social media, and at conferences, such as the NCD symposium hosted by the South African CEBHA + partners in March 2020 [48] or the Joint Annual Scientific Conference in Uganda.

**Table 4:** Overview of modes of delivery

	Ethiopia	Malawi	Uganda	Rwanda	South Africa
Face to face meetings	●	●	●	●	●
Virtual meetings	●		●	●	●
Phone calls/ messaging	●	●	●	●	●
Webinars					●
Twitter and other social media	●		●	●	●
Conferences	●		●	●	●
Policy dialogues	Planned	●		Planned	●

### Item 7: Where was IKT implemented?

Most teams engaged with stakeholders in the capital of the respective country. In Ethiopia, the focus was primarily on Addis Ababa and Adama, but other stakeholders had connections in various regions, including Jimma, Dire Dawa, and Bahir Dar. The Malawi team interacted with stakeholders in the country's two main cities, Blantyre and Lilongwe. In Rwanda, stakeholders were based in the Burera district in the Northern province and in Gasabo district in the capital Kigali. Ugandan stakeholders were situated in Kampala, and South African stakeholders were based in Cape Town and Pretoria. In Table 5, we describe the research focus of these eight institutions, their institutional KT infrastructure, as well as KT structures and regular activities beyond the institution, including broader EIDM related capacity-building and networks.

**Table 5:** Characteristics of CEBHA + institutions, institutional and macro KT and IKT context

Name of the institution (abbreviation), department; City, Country; CEBHA + research tasks	Institution’s NCD research focus	Institutional KT infrastructure, KT structures and regular KT activities beyond the research institution, capacity-building, networks
Armauer Hansen Research Institute (AHRI), NCD research directorate Addis Ababa, Ethiopia Research tasks: RT1, RT2	*Research focus:* Cancer research, NCD risk factors, podoconiosis and air pollution *Further information:* Funding sources are the Swedish, Norwegian and Ethiopian governments	**Institutional KT infrastructure** - The AHRI Knowledge management directorate has goals that overlap with CEBHA +’s, which led to joint activities; support for networking and capacity-building activities - AHRI’s CEO is regularly involved in round table discussions at MoH **Broader KT structures and activities** - The Ethiopian Public Health Institute (EPHI) through its KT directorate is building the culture of evidence generation, synthesis and utilisation by delivering different capacity-building training to different stakeholders and partners. EPHI is mandated to conduct systematic reviews, issue briefs, policy briefs that will be utilised for the decision-making process at the MoH **Capacity-building** - EPHI is delivering different training and mentorship support planned by CEBHA + initiatives - Multiple existing EIDM short courses organised by other parties **Networks** Existing network of organisations and individuals interested in IKT and EIDM in the country
Kamuzu University of Health Sciences (KUHeS), Global and Public Health Department Blantyre, Malawi Research tasks: RT3	*Research focus*: Broad range of public health research	**Institutional KT infrastructure** - The Research Support Centre at KUHeS organises annual research dissemination conferences which are attended by policy-makers **Broader KT structures and activities** - The Research Unit at the MoH regularly invites researchers to jointly work on policy briefs, which includes a prioritisation exercise and building communities of practice. Finalised policy briefs may be discussed in policy dialogue or parliamentary committee - Malawi KTP ‘s main function is to promote evidence-informed health policy. It provides a coordinated approach to the generation and utilisation of health research. KTP Malawi facilitates research dialogue between CEBHA researchers and policy-makers - MEIRU researchers are commonly invited to technical working groups **Networks** - There are further networks fostering EIDM work in the country, e.g. the EVIDENT network aiming to enhance research responsiveness to policy needs - Health Economics Policy Unit (HEPU) policy lab is a platform where key stakeholders develop research proposals, implement research and process evidence into policy - AFIDEP focuses on strengthening the capacity in evidence use for decision-making and providing evidence for technical assistance to increase progress towards the sustainable development goals Overall small public health community in the country, some researchers with previous policy-making expertise and vice versa which facilitates collaboration
Malawi Epidemiology and Intervention Research Unit (MEIRU) Lilongwe, Malawi Research tasks: RT1, RT2	*Research focus*: NCDs	
University of Rwanda (UR), College of Medicine and Health sciences Kigali, Rwanda Research tasks: RT1-4	*Research focus*: Broad range of health and public health research	**Broader KT structures and activities** - Regular exchanges with Planning, M&E and Health Financing Departments at Rwanda MoH - Close collaboration with the health implementation body (Rwanda Biomedical Center (RBC)), boundary spanner roles between UR and RBC **Networks** Strong movement for citizen science and research co-production approaches, involving knowledge users and communities
Chronic Disease Initiative for Africa (CDIA), Department of Medicine, University of Cape Town Cape Town, South Africa Research tasks: RT1, RT2	*Research focus:* Development and evaluation of models of chronic disease care and prevention	**Broader KT structures and activities** - Involved in consultation rounds related to the new National Strategic Plan for NCDs and Obesity Prevention - Representative from the provincial DoH attending CDIA management committee meetings **Networks** Network of multidisciplinary researchers and policy-makers which serves as a regional hub
South African Medical Research Council, Cochrane South Africa (CSA) Cape Town, South Africa Research tasks: RT1-3, RT5	*Research focus:* Evidence synthesis, evidence-informed decision-making and knowledge translation	**Institutional KT infrastructure** - CSA and SU’s CEBHC are dedicated to evidence synthesis, knowledge translation, and promoting the uptake of current best evidence in healthcare policy and practice in Africa Broader KT structures and activities - Long-standing collaboration between CSA and SU working with the same stakeholders, in particular through guideline work and providing systematic reviews for the SA government as well as previous projects linking researchers and decision-makers - CSA: Regular contribution to expert committees as evidence experts for national and provincial departments of health **Capacity-building** - SU: regular courses on EIDM and knowledge translation: ‘*Evidence-Informed Decision-Making: The Art, Science and Complexity of (Integrated) Knowledge Translation*’ and ‘*Engaging with Decision-makers: Issue Briefs for policy and practice*’ for students, researchers, public health practitioners and policy-makers (Jessani, Hendricks 2022)) **Networks** - CSA is part of the global, independent Cochrane network of researchers, professionals, patients, carers and people interested in health. Cochrane is a non-profit organisation that prepares and disseminates information (in the form of reviews) on what works and what doesn’t in healthcare. These reviews enable policy-makers, health service providers and the public to make informed decisions The CEBHC and CSA jointly manage and lead the SA GRADE Network, which aims to advance the learning about and use of GRADE for evidence-informed decision- making for policy and practice in South Africa
Stellenbosch University (SU), Centre for Evidence Based healthcare (CEBHC) within the Division of Epidemiology and Biostatistics, Department of Global Health Stellenbosch, South Africa Research tasks: RT2-3, RT5	*Research focus:* Evidence-based healthcare *Further information:* The Dept. of Global Health aims to address complex health and social problems and to advance health equity	
Makerere University (MU), School of Public Health, Department of Disease Control and Environmental Health, Trauma, Injuries and Disabilities Unit Kampala, Uganda Research tasks: RT4	*Research focus:* Prevention of road traffic injuries, mobility and health	**Institutional KT infrastructure** - The unit undertakes regular research dissemination workshops **Broader KT structures and activities** - Unit member was co-opted onto the National Road Safety Committee - CEBHA + researchers contributed evidence for the parliamentary committee concerned with road traffic injuries (through the policy briefs and dissemination meetings) and participated in drafting the National Road Safety Action Plan **Networks** The institution has a strong reputation and is perceived as an authority in research and health topics

MoH = Ministry of Health, DoH = Department of Health, KTP = Knowledge Translation Platform, EIDM = Evidence-Informed Decision-Making, CEO = Chief Executive Officer; **Research tasks (RT)—RT 1:** Evidence-informed policies and practices on screening approaches for hypertension and diabetes, and those at high risk of cardiovascular disease in sub-Saharan Africa, **RT 2:** Evidence-informed policies and practices on integrated models of healthcare delivery for hypertension and diabetes in sub-Saharan Africa, **RT 3**: Evidence-informed policies and practices on population-level interventions to prevent diabetes and hypertension in sub-Saharan Africa, **RT 4:** Finding the evidence for improved implementation of road traffic injury prevention interventions, **RT 5:** Promotion of an integrated, rigorous methodological approach across CEBHA + research tasks and activities

#### KT culture

Overall, stakeholder and/or decision-maker involvement was common practice at multiple sites and at times mandatory, e.g. for seeking research approval from relevant authorities and/or coordinating research activities. We describe the respective culture of knowledge exchange in more detail in Box 1.

Box 1: culture of knowledge translation In the context of the three **South African** institutions, doing research with the goal of having an impact on decision-making is at the “heart” of what the research institutions aim to do. For example, the involvement of stakeholders is taught as standard practice for conducting systematic reviews at one institution and students take on research questions prioritised by decision-makers for their Master’s theses in public health. Public health specialists at the Western Cape Provincial DoH regularly engage with researchers to access evidence. DoH policy, for example with respect to health programmes for the Khayelitsha township, is based on local research evidence. In **Malawi**, an existing knowledge translation platform offered an important opportunity for continuous interaction with decision-makers in the MoH. MEIRU always works with policy-makers to decide research priorities, this is facilitated by boundary spanners working at the MoH and in research roles. The **Rwandan** team reported great interest among researchers and decision-makers regarding capacity-building for EIDM. The **Ethiopian** team reported a gradual uncovering of dedicated existing and emerging KT networks in the country and at their institution over the course of the project. In **Uganda**, the CEBHA + team drew on a rich network of existing relationships with a diverse range of stakeholders in the road traffic injury prevention space.

#### COVID-19 context

As the COVID-19 pandemic emerged, CEBHA + researchers engaged in further processes and structures to facilitate decision-makers’ access to COVID-19 related research. These activities were organised at the level of CEBHA + institutions and included working on rapid reviews and guidelines on prioritised COVID-19 topics (CSA, SU), building the capacity of researchers to communicate research findings to decision-makers (KUHeS), providing expert advice or serving on expert committees to inform government policy (CSA, SU), responding to ad hoc requests for input from decision-makers (CSA, SU, UR), and providing a platform to disseminate COVID-19 research findings (KUHeS, CSA). Other sites conducted primary research to inform decision-making, for example on COVID-19 vaccine hesitancy (CDIA); the impact of lockdown on mobility patterns (MU); and, through a SARS-CoV-2 serological test validation study and a study on the impact of COVID-19 on diabetes care and management (AHRI).

### Item 8: When and how much?

IKT implementation in some sites started even before IKT strategies were formalised in early 2019, including when decision-makers participated in the priority-setting exercise for defining research topics in 2013 [25]. Further opportunities for engagement were present at all stages of the research process, including the development of research questions, methods and protocols; research approval and planning; data collection; data interpretation; write-up; and dissemination. The actual timing and intensity of IKT activities depended on stakeholder preferences, IKT teams’ resources and capacity as detailed in the IKT strategies, and contextual circumstances for more ad hoc interactions.

### Item 9: Tailoring of IKT strategies

IKT strategies were expected to provide an initial plan for stakeholder engagement tailored to individual stakeholder needs and context that would subsequently be adapted as the project progressed. All country teams considered the level of tailoring of their IKT strategies to individual stakeholders as having occurred to a medium or large extent, both at the onset of and throughout the project. Main reasons for tailoring IKT strategies were related to (i) stakeholders, (ii) the project itself, or (iii) macro-level changes.

Adjusting to individual stakeholder’s preferences, for example regarding preferred communication modes or interest, but also attention to government protocol and pre-existing relationships were named as reasons for tailoring initial IKT strategies. Stakeholder turnover and identifying new stakeholders presented reasons for tailoring as the project progressed. The South African team noted the relevance of choosing appropriate and constant messengers for communicating with priority stakeholders. The Ugandan team had already undertaken substantial stakeholder engagement before the IKT strategy was developed, hence fitting the strategy retrospectively. In Malawi and Rwanda, delays in conducting the planned research led to project-related tailoring of IKT activities. In Ethiopia, the IKT strategy and CEBHA + research scope were adjusted substantially to avoid duplication of research efforts after an overlapping research activity was identified. In Rwanda, Uganda, and South Africa, tailoring was prompted by changes in the macro context (i.e. parliamentary elections). All sites adjusted IKT activities substantially due to the COVID-19 pandemic.

### Item 10: Modifications of the IKT approach

Three major modifications of the IKT approach and IKT strategies occurred throughout the implementation period. These included (i) frequent ad hoc engagements to complement planned engagements, (ii) broadening prioritised IKT partners to include stakeholders beyond decision-makers, and (iii) introducing more feasible monitoring processes.

#### Ad hoc engagements

In addition to the planned stakeholder engagements, as detailed in the IKT strategies, country teams leveraged ad hoc interactions as further engagement opportunities particularly to provide decision-makers with evidence during the COVID-19 pandemic or related to national strategic documents [31]. Hence, ad hoc engagements covered topics both within and beyond the scope of the CEBHA + project. Some of these constituted one-off contacts with decision-makers, whilst other engagements led to long-term interaction, prompting (formal or informal) inclusion of the respective decision-maker in IKT strategies. The ad hoc engagements leading to continuous engagement thus constituted instances of intervention tailoring. On the other hand, we conceptualise one-off ad hoc engagements complementing planned, continuous engagements as modifications of the IKT approach because they do not meet the IKT characteristics of long-standing interaction to build lasting partnerships.

#### Breadth of prioritised IKT partners

The five IKT strategies targeted a diverse range of decision-makers and other stakeholders, reflecting the range of research activities in the project. All sites prioritised decision-makers at the respective MoHs. Further priority stakeholders were identified in other ministries, local authorities, healthcare institutions, or in the UN system (Table 6). Beyond these groups, CEBHA + teams engaged a broader range of stakeholders, including non-governmental organisations (NGOs) involved in prevention and awareness-raising about NCDs and road traffic injuries, local communities, and academic stakeholders.

**Table 6:** Overview of prioritised stakeholders and stakeholder groups

	Ethiopia	Malawi	Rwanda	South Africa	Uganda
International stakeholders		WHO		WHO	
National MoH	●	●	●	●	●
Other Ministries			Ministry of Sports, Ministry of Agriculture and Animal Resources, Ministry of Public Service and Labour, Ministry of Infrastructure		Ministry of Work and Transport
Local authorities	Ethiopian Public Health Institute		Rwanda Biomedical Centre (MoH implementing agency), road traffic police department	Provincial Departments of Health (DoH)	Kampala Capital City Authority, road traffic police dept
Healthcare institutions	●		●	●	●
NGOs	●	●	●	●	●
Other stakeholders	Communities, academic stakeholders		Communities	Research council	

#### Feasible monitoring processes

The IKT approach initially included an interaction logbook to monitor IKT activities [34]. Monitoring indicators included, for example, the number of meetings held with a specific stakeholder, the number of decision-makers participating in meetings, decision-maker requests, and email replies. However, this proved too impractical and instead of using the bespoke logbook, monitoring methods were diverse across implementation sites: In Ethiopia, Uganda and South Africa, IKT focal points used the stakeholder engagement matrix for monitoring purposes. In South Africa, team discussions constituted an important aspect of monitoring that informed the updating of the IKT strategy. At all sites, further site-specific approaches to monitoring were undertaken (e.g., notes, document collection, spreadsheets, and reporting in the quarterly IKT update).

### Item 11: How well was the IKT approach implemented?—planned

Across the CEBHA + consortium, assessment of overall intervention adherence and fidelity were planned as part of the IKT evaluation [34]. At the five implementing sites, adherence and fidelity to the initially specified IKT strategies were recognised to likely vary depending on contextual circumstances, e.g. staff continuity or stakeholder responsiveness. Hence, adaptations to the IKT strategies were anticipated (“tailoring”) and were planned to be documented in IKT strategy updates.

### Item 12: How well was the IKT approach implemented?—actual

Adherence and fidelity were primarily investigated for (i) the implementation of *continuous* stakeholder engagement across the five sites and (ii) the utilisation of IKT strategies, including the development, updating and monitoring of the strategies.

#### Continuous stakeholder engagement

Across the research consortium, continuous stakeholder engagement was adhered to and implemented with a high degree of fidelity.

Stakeholder involvement in the research process peaked for activities early and late in the process, demonstrating continuity of engagements. All sites mentioned stakeholder engagement in the selection of the research topic and the concrete research questions. All sites, except Rwanda, reported little or no stakeholder involvement in data collection; but stakeholders were frequently reported to facilitate data collection, except in South Africa. Data analysis with stakeholders was rarely mentioned but joint data interpretation, publication, and reporting were more common. Except for Ethiopia—where this was planned at the time of writing this article—dissemination of research outputs systematically involved CEBHA + stakeholders. Actual implementation of research results by stakeholders was rarely mentioned, except by interview participants in Uganda.

In the early-stage IKT evaluation, survey respondents reported a high frequency of interaction. Almost half of the researchers indicated that they had participated in at least five face-to-face meetings with their respective partners (11/23, 48%). Among stakeholders, two out of seven (29%) indicated that they had participated in at least five meetings, with the difference in frequency resulting from multiple stakeholder contacts per researcher. Researchers had also engaged more often via email [> 6 times since the beginning of the project: 13/24 (54%) of researchers vs. 2/7 (29%) of stakeholders], by phone [> 6 times: 14/24 (58%) vs. 2/7 (29%)], through messengers such as WhatsApp [> 6 times: 10/24 (42%) vs. 1/7 (14%)], and via social media [> 6 times: 5/24 (21%) vs. 1/7 (14%)]. Six out of seven decision-makers stated that they had reached out to CEBHA + researchers at least once for expert advice (86%) and the majority of researchers had been approached for advice by their respective partners at least once (76%).

#### Development, updating and monitoring of IKT strategies

**Fig. 2:**
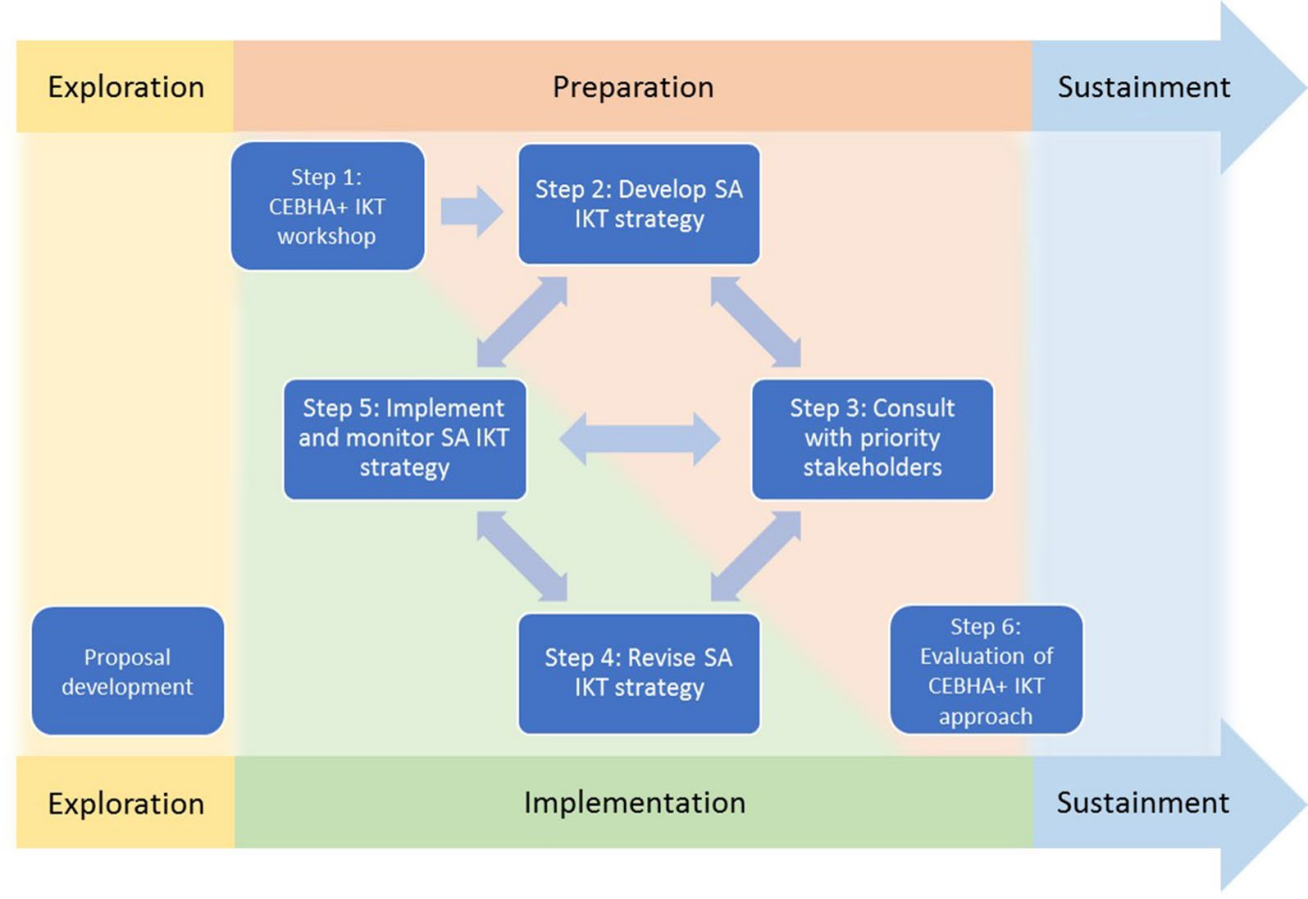
Revised visualisation of the CEBHA + IKT approach [30]

The four steps for developing IKT strategies as detailed under item 4 (Fig. 1) were undertaken by all sites, resulting in five initial IKT strategies. The IKT team in South Africa undertook a structured reflection that indicated that the process of developing an IKT strategy was much more iterative and dynamic than conceptualised in the IKT approach, as informed by the EPIS framework and visualised in Fig. 2 [30].

Data on the extent of monitoring and subsequent tailoring reported by most sites was contradictory: Responses in the reflective survey indicated that monitoring and updating of the IKT strategy took place, which contrasts with the document analysis that indicated that Excel-based IKT strategies were rarely updated. One evaluation participant noted that “little attention” was paid to monitoring and updating of IKT strategies after these were finalised and that most engagements happened ad hoc. Hence, while IKT teams did adapt their approach to stakeholder engagement based on local experience and adjusted to new opportunities in an informal manner, these adaptations were not documented in updated, formal IKT strategies.

## Discussion

### Summary of results

This article describes how IKT was planned, implemented and monitored in an international, NCD- and EIDM-focused research consortium. Building on pre-existing stakeholder networks and KT practices, five African country-specific IKT strategies for a broad range of stakeholders were developed and tailored to specific CEBHA + research activities, stakeholder preferences, local context, team capacities, and emerging opportunities. Implementation of IKT strategies included diverse IKT activities and required further tailoring and broader modification of the approach. Monitoring and updating of IKT strategies was conducted less formally than initially planned. Despite major disruption by the COVID-19 pandemic continuous collaborations with stakeholders were established or continued and, indeed, presented new opportunities for IKT.

### IKT evaluation literature

To our knowledge, this is one of the first studies attempting to describe the development, implementation and monitoring of IKT strategies implemented across multiple countries using an established checklist. This work was motivated by our realisation, at the onset of the CEBHA + consortium, that few descriptions of IKT interventions existed and that interventions were poorly reported [3], which continues to be a challenge [49, 50]. Over the last few years, however, more descriptions and evaluations have become available. Process and outcome evaluations to examine IKT in rehabilitation [51], occupational disease [52], violence prevention [53], health-promoting schools [54], and healthcare [55, 56] provide key insights. Protocols for research evaluating IKT approaches in disability participation [57] and in rehospitalisation prevention [58] are also available. We believe that the present paper adds significantly to the literature by sharing practical lessons from IKT development and implementation of a standardised yet tailored approach across five countries.

### IKT activities and tailoring

Our work illustrates a multiplicity of IKT activities. IKT with policymakers, as shown in a scoping review of IKT approaches in public health, involves knowledge users at all stages of the research process using a range of IKT activities, where “different methods of engagement serve different functions” [10]. This is reflected in CEBHA + , where—although the overarching rationale was to increase the use of contextualised evidence in decision-making—site-specific IKT strategies serving a broader range of objectives were put in place. These objectives called for the involvement of different groups of knowledge users through diverse activities, at varying levels of involvement. The CEBHA + IKT approach hence embraced what has been described as the “continuum of co-production”: IKT processes are “situated along a continuum in terms of the number of research stages, the way stakeholders are involved in co-production, the project scope and scale, and the degree of adherence to the principles and practice of co-production achieved” [15]. Some interactions, which we will explore in more depth in our IKT evaluation, may more appropriately be called research participation or consultation, representing a shift away from the goal of co-production, which has also been observed in other studies [16].

Our approach of careful tailoring of IKT strategies to local circumstances and capacities, research activities, and stakeholder preferences contrasts with the lack of tailoring described in KT practice in some low and middle-income countries (LMICs) [59]. These differences likely arise due to CEBHA +’s dedicated funding for IKT, particular emphasis on understanding context through stakeholder analysis, the definition of tailored outputs as project deliverables, and an explicit, ongoing focus on tailoring in IKT training and meetings.

### Modifications to the CEBHA + IKT approach

The first of three modifications of the IKT approach, ad hoc engagements complementing the systematically planned IKT activities [31], points to one challenge of designing IKT strategies a priori. These IKT strategies may occasionally appear too static or impractical for use in a dynamic stakeholder landscape, hence resulting in IKT strategies being filed away. In our work, whilst this has occurred in some cases, there have also been instances of substantial updating of IKT strategies. In our experience, the benefits of developing and using IKT strategies outweigh the required effort and resources. These benefits include but are not limited to the following: stakeholder analysis allowing for a better understanding of the research, policy, and practice context [29, 60]; improved team communication about existing relationships and partnerships; clarity about the aims of stakeholder engagement [28]; striking a balance between cultivating existing relationships, building new strategic partnerships, and responding to ad hoc requests; and adequate allocation of resources. At the same time, responsiveness to decision-maker queries constitutes a key mechanism for building relationships [61] and exploitation of other opportunities using the much-cited ‘policy window’ presents another key strategy for influencing policy changes [62]. In future IKT work, we would therefore recommend conceptualising IKT strategies as flexible tools that are highly compatible and complementary with researcher responsiveness to ongoing and ad hoc stakeholder engagement.

Secondly, the monitoring process was modified as the pre-designed tools for monitoring IKT activities proved less helpful than anticipated and were resource-intensive. This resulted in a mix of monitoring efforts, some of which were very informal. Monitoring relationships between two individuals, two organisations, or between an individual and an organisation is undoubtedly challenging. Therefore, the identification of useful and informative indicators and monitoring processes should be a focus of future research. Whilst a substantial list of these indicators has been proposed [63], pragmatic approaches to capturing these need to be developed. These could for example integrate routine data collected for other purposes.

The third modification of the IKT approach occurred with respect to the range of knowledge users prioritised for IKT activities. Whilst IKT has been defined as involving decision-makers [1], CEBHA + teams included individuals beyond the decision-making sphere. These “others” comprised three groups: NGOs, other academics, and communities. NGOs play a particular role in KT in LMICs as they take on a range of activities at the nexus of research, policy and practice, including as knowledge brokers and implementers, and in the research process, including in research priority setting, resource mobilisation, and undertaking operational research [26]. Prioritisation of NGOs in IKT strategies hence makes sense as part of a multi-tiered, strategic effort to enhance evidence use in healthcare and public health decision-making. Others have demonstrated how these alliances influenced decision-making, albeit in a Canadian context [64].

Other academics were prioritised for IKT activities in CEBHA +, e.g. for joint capacity-building with decision-makers and university students. Whilst this group may rarely be conceptualised as decision-makers they may have a profound influence on decisions regarding healthcare and public health education, therefore strengthening EIDM in the mid- to longer term [29]. Overall, these experiences point to the relevance of considering groups beyond traditional decision-makers in future IKT work.

### Consideration of context

One major change of contextual circumstances during our study occurred with the COVID-19 pandemic, putting most IKT activities on a temporary hold from March 2020 onwards and requiring substantial tailoring of activities. While the pandemic paused some NCD-focused engagement, it provided an opportunity to enhance KT exchanges between decision-makers and researchers on COVID-19 topics, both in CEBHA + and around the globe. Research on pandemic-related policy advice has illustrated some of the challenges researchers face in these advisory roles [65] and some claims of ‘following the science’ have been identified as performative scientism [66]. Since these phenomena are not specific to the COVID-19 pandemic but may also occur regarding to other health emergencies or political developments, we suggest that such aspects be incorporated in IKT training.

IKT is taking place in a multi-layered context, which is of particular interest to researchers aiming to investigate IKT processes and partnerships [4, 15, 26, 67]. Our stakeholder mapping and analysis sought to generate a starting point for understanding the respective contexts which vary substantially regarding the existing KT structures, processes, and capacities (Table 5); through continuous interaction with decision-makers we further aimed to improve our understanding of their realities, priorities, and the decision-making processes. Existing EIDM structures and networks presented an important context for our work which we aimed to connect to. This included, for example, the Malawi team’s interaction with an existing KT platform [68] and the Ethiopian team’s collaboration with the Ethiopian Public Health Institute’s KT directorate. We recommend that IKT implementers identify and utilise these structures for (I)KT activities to avoid duplication of structures and efforts.

### Use of the TIDieR checklist

As suggested by Hoekstra [50], we chose the TIDieR checklist to report on our IKT ‘intervention’ to enhance the consistency of reporting [35]. We hope that this can improve the transferability and reproduction of IKT in different contexts and projects [69]. This presents a challenge for the IKT research field in which IKT activities are understood as *complex interventions in complex systems* [33]. We also found it difficult to define which part of the IKT approach constitutes the ‘intervention’, as different IKT activities, the formal planning of IKT activities, and/or each individual relationship between a researcher and a stakeholder of interest could be conceptualised as an IKT intervention. We made a decision to describe the CEBHA + IKT approach at the cross-site and site-level but not in depth at the activity and stakeholder level, but acknowledge that this would have likely illustrated the extent of tailoring of the intervention in more detail [70].

### Beyond the TIDieR checklist: barriers and facilitators of IKT

One aspect that we do not address in this article are the factors facilitating IKT, as we focused on TIDieR items. Indeed there is a rich 'barriers and facilitators’ literature on IKT, as documented in various scoping and other reviews: These examine KT barriers and facilitators in African health systems [49, 71], in health policy dialogue in African countries [72], in LMICs [59], in healthcare [3], and with public health policy-makers [10]; as well as reviews of evidence use by policy-makers [73], and for engaging knowledge users in evidence synthesis [74]. What these articles highlight is the need for capacity-building and ensuring sufficient skills in IKT and EIDM. IKT in particular requires a broad set of both technical and interpersonal skills [75]. However, current education and training programmes for health professionals rarely include courses that train (future) researchers and policy-makers in the co-production of evidence. In the African context, a plethora of one-off courses are available, whilst institutionalised or university-based courses are scarce [29]. In LMICs, capacity strengthening initiatives tend to focus more on individuals than organisational or institutional strengthening [26, 59]. CEBHA + has aimed to address this gap by developing and implementing a course on evidence-based public health and integrating it into institutional curricula.

Overall, our IKT approach required substantial investment of time and resources, as described by many others [10]. Along with the necessity to be attuned to the unintended effects of IKT and other co-production approaches, this emphasises the need for rigorous evaluation [14, 76]. This is further underscored by the inherent contradiction that although KT and IKT advocates set out to improve EIDM, whilst embarking on KT and other partnership approaches, the evidence for impacting health outcomes is sparse [14, 50]. We hope to address this issue with our process and outcome evaluation [34].

### Reflexivity

The IKT team, i.e. the individuals involved in developing, implementing, monitoring and evaluating IKT in CEBHA +, form a heterogeneous group with different levels of expertise, experiences, cultural backgrounds, and tacit knowledge regarding stakeholder engagement, and many views on IKT. As a team we tried to understand, consider, reflect upon and reconcile this diversity throughout the CEBHA + project and in the write-up of this manuscript. This has some implications including the following: The allocation of tasks and funds determined at the initial stages of project planning envisioned the implementation of IKT at the five African sites and setting up a semi-external IKT evaluation with evaluators from Germany. CEBHA + researchers based at LMU Munich, Germany, also led the research task subsuming these activities (see Additional file 1) with strong support from KT experts at SU and CSA, South Africa. Hence, the approach was driven by individuals from one high-income and one upper-middle-income country and implemented in four low-income countries and South Africa. We have accounted for some potential contextual differences, e.g. by grounding the IKT training in an established course focused on tailoring stakeholder engagement to contextual circumstances and stakeholders in LMICs [29]. However, we have not been able to address other challenges, in particular related to the power dynamics that come with the position of the evaluator and those being evaluated, as well as issues of foreign ‘gaze’ and ‘pose’ [77]. In this paper, we have aimed to reconcile the foreign and local perspectives and positions. However, as is reflected in the authorship order of this paper, we may have only achieved this partly and it is likely that some remnants of these aspects still shaped parts of this work.

### Strengths and limitations

This study has several limitations. With respect to the study design, whilst this body of work integrates some process and outcome evaluation elements, it does not constitute an evaluation per se but a structured description and reflection of our CEBHA + IKT approach. Of note, the IKT evaluation that also informed this paper constitutes a longitudinal, not an experimental evaluation, hence limiting claims of causality about observed phenomena. With respect to the data collected, analysed and presented here, we were likely unable to capture all of the very broad and diverse set of IKT activities implemented in CEBHA in detail, partly due to the monitoring challenges outlined above. Our description represents our subjective perspective on the IKT approach as relatively limited data were available from CEBHA + policy and practice partners. Furthermore, for some sites, we were unable to include the perspective of the initial IKT focal points due to staff turnover. This may have led to biased reflections about the development and initial implementation of IKT strategies and may have impacted on the continued implementation and monitoring of these strategies.

Nevertheless, this study integrates a variety of data sources and, by representing the perspectives of most individuals involved in developing, implementing and monitoring IKT in CEBHA + throughout its project lifespan, the article paints a nuanced, realistic picture of how IKT was planned and undertaken. The utilisation of the TIDieR checklist, whilst not ideal for describing an IKT intervention, presented a helpful structure to organise key characteristics of our approach. The process of writing this paper provided an opportunity for CEBHA + researchers to discuss and reflect on IKT activities and distil lessons for future work. These team discussions also helped to address some of the limitations outlined above, in particular, they helped to make sense of the volume of data and variety of perspectives. The paper further lays the groundwork for reporting the CEBHA + IKT evaluation results.

## Conclusion

Whilst more descriptions and evaluations of IKT are becoming available, this paper addresses a relevant evidence gap as there are few in-depth accounts of how IKT approaches are implemented and, notably, across multiple countries. Our detailed description of the tailored IKT strategies, activities, and contextual characteristics related to EIDM in Ethiopia, Malawi, Rwanda, South Africa and Uganda provide practical guidance for others embarking on IKT activities and contribute to the unpacking of the IKT ‘blackbox’. Based on our experiences, we recommend that IKT implementers (i) undertake thorough and repeated stakeholder analysis to examine existing KT and EIDM infrastructure and networks for identifying partners for collaboration; (ii) consider involving more ‘atypical’ decision-makers, such as NGOs, in particular in settings where NGOs are an important actor in healthcare and public health; (iii) tailor and monitor IKT activities to the needs and possibilities of different sites; (iv) ensure there are resources earmarked for IKT; (v) and be mindful of team capacity and time for IKT. Future IKT-focused research is needed on identifying and testing feasible and informative practices for monitoring IKT activities and subsequent updating of IKT strategies.

### Supplementary Information

**Additional file 1.** Overview of CEBHA + research tasks and activities.

**Additional file 2.** Reflective Survey and corresponding TIDieR checklist items.

## Data Availability

The datasets generated and/or analysed during the current study are not publicly available due to the small number of responses and potentially identifiable information. Subsets of the datasets may be made available from the corresponding author on reasonable request.

## Abbreviations

CEBHA +: Collaboration for Evidence-based Healthcare and Public Health in Africa
IKT: Integrated knowledge translation
KT: Knowledge translation
EIDM: Evidence-informed decision-making
NCD: Non-communicable diseases
MoH: Ministry of Health
NGO: Non-governmental organisation
SU: Stellenbosch University
AHRI: Armauer-Hansen Research Institute
LMU Munich: Ludwig-Maximilians-Universität München
MU: Makerere University
CSA: South African Medical Research Council, Cochrane South Africa
KUHeS: Kamuzu University of Health Sciences
MEIRU: Malawi Epidemiology and Intervention Research Unit
UR: University of Rwanda
TIDieR: Template for intervention description and replication
LMIC: Low and middle income countries

## Footnotes

1 In this article, we commonly refer to IKT policy and practice partners as ‘stakeholders', which is the terminology that became established in CEBHA + , as opposed to ‘decision-maker’ or ‘knowledge user’. This reflects the fact some of the partners are less commonly understood to be decision-makers, e.g. university teaching staff, as we describe below.
