# Supplementary material for: Developing, implementing, and monitoring tailored strategies for integrated knowledge translation in five sub-Saharan African countries
Source: Health Res Policy Syst. 2023 Sep 4;21:91. doi: 10.1186/s12961-023-01038-x (PMC10478471; doi:10.1186/s12961-023-01038-x)
Supplement: Supplementary file 1 — Additional file 1. Overview of CEBHA + research tasks and activities. [file 12961_2023_1038_MOESM1_ESM.pdf]

Overview of CEBHA+ research tasks and activities

**Research task 1: Evidence-informed policies and practices on screening approaches for hypertension and diabetes, and those at high risk of cardiovascular disease in sub-Saharan Africa**	
1.1	Review of existing evidence on the effectiveness of different screening approaches for hypertension and diabetes in low- and middle income countries (LMICs)
1.2	Conduct a robust evaluation of the use of the total non-laboratory CVD risk score
1.3	Prepare evidence-informed policy briefs and have policy dialogues
**Research task 2: Evidence-informed policies and practices on integrated models of healthcare delivery for hypertension and diabetes in sub-Saharan Africa**	
2.1	Determine the prevalence of diabetes, hypertension and depression amongst HIV-positive people receiving Antiretroviral Therapy (ART)
2.2	Review existing evidence on integration of care for hypertension and diabetes
2.3	Review existing evidence on integration of care for other diseases
2.4	Prepare evidence-informed policy briefs and have policy dialogues
2.5	Implement a comprehensive approach to patient education and counselling for patients with non-communicable chronic diseases in primary care
**Research task 3: Evidence-informed policies and practices on population-level interventions to prevent diabetes and hypertension in sub-Saharan Africa**	
3.1	Undertake a systematic review of the effectiveness of population-level interventions to prevent diabetes and hypertension in low- and middle-income countries
3.2	Conduct a situational analysis to identify population-level interventions currently being implemented in Malawi, South Africa and Rwanda
3.3	Evaluate the implementation of two selected ongoing population-level interventions targeting risk factors for diabetes and hypertension, one in Rwanda and one in either Malawi or South Africa

3.4	Prepare evidence-informed policy briefs and have policy dialogues and stakeholder engagement with policy-makers and healthcare professionals
**Research task 4: Finding the evidence for improved implementation of road traffic injury prevention interventions**	
4.1	Collect and assess the quality of currently available data on the epidemiology of road traffic injuries
4.2	Identify key policy and programmatic aspects in the design, implementation and evaluation of existing interventions to reduce the incidence of pedestrian road traffic injuries
4.3	Review the effectiveness of international pedestrian-targeted interventions to prevent the occurrence of road traffic injuries
4.4	Evaluate the implementation of current pedestrian-targeted interventions to prevent occurrence of road traffic injuries in Rwanda and Uganda
**Research task 5: Promotion of an integrated, rigorous methodological approach across CEBHA+ research tasks and activities**	
5.1	Implement an integrated approach from research conceptualisation to implementation of findings that engages decision-makers throughout the research process
5.2	Ensure highest possible scientific rigour for all types of research conducted under CEBHA+
5.3	Evaluate enhanced competence of CEBHA+ researchers and higher-quality research output
5.4	Assess added value of the CEBHA+ research co-production approach
