# Supplementary material for: Developing, implementing, and monitoring tailored strategies for integrated knowledge translation in five sub-Saharan African countries
Source: Health Res Policy Syst. 2023 Sep 4;21:91. doi: 10.1186/s12961-023-01038-x (PMC10478471; doi:10.1186/s12961-023-01038-x)
Supplement: Supplementary file 2 — Additional file 2. Reflective Survey and corresponding TIDieR checklist items. [file 12961_2023_1038_MOESM2_ESM.pdf]

Additional file 2: Reflective Survey

**TIDieR checklist item**	**Description**	**Corresponding question in Survey**
Item 1: Brief name		
Item 2: Why?	Rationale, theory, or goal of the elements essential to the intervention	What was the rationale you and your colleagues had in mind when developing the site specific IKT strategy? (late 2018/early 2019)
Item 3: What?	Materials used in the intervention and in the training of intervention providers	Procedures: Please select the respective steps you and your colleagues pursued and/or participated in when developing, implementing and monitoring your IKT strategy. Check all that apply.
		Materials: Please select all materials you used to DEVELOP and UPDATE your IKT strategy
		Please add any other materials you used to DEVELOP and UPDATE your IKT strategy
		Please select all the materials you used to MONITOR your IKT strategy.
		Please add any other materials you used to MONITOR your IKT strategy
		Please feel free to add other steps you undertook when developing or updating the IKT

		strategy.
		Feel free to share any reflections or insights about the development or updating of the IKT strategy.
Item 4: Who provided	For each category of intervention provider (such as CEBHA IKT Focal Points, CEBHA+ researchers, CEBHA+ PIs/Co-PIs), describe their expertise, background, and any specific training given	Provider: Who engaged with stakeholders (i.e. implemented the IKT strategy) at your site?<br>Check all that apply.
		Provider: Who else engaged with stakeholders at your site?
		Provider: Which expertise regarding IKT did the IKT focal point have?
		Provider: Which expertise regarding IKT did the other people who engaged with stakeholders have (e.g. previous experience in stakeholder engagement)?<br>Please indicate a team average.
		Provider: In your team, which training did the persons who engaged with stakeholders receive (e.g. IKT workshop, issue brief workshop, online resources)? Check all that apply.
Item 5: How?	Describe the modes of delivery (such as face to face or by some other mechanism, such as internet or telephone) of the intervention and whether it was provided	By which mode did you interact with stakeholders at your site? Please select all that apply.
		Please add any other modes you used to interact with stakeholders at your site.
		How did you interact with your CEBHA+ colleagues at your site? Please select all that apply.

	individually or in a group	Please add any other modes of how you interact with CEBHA+ colleagues at your site.
Item 6: Where?	Types of Locations where the intervention occurred, including any necessary infrastructure or relevant features	Please indicate the city, cities or regions in which the IKT strategy was implemented
		Please indicate if there was relevant (I)KT infrastructure available that you used to implement your IKT strategy (e.g. national KT platform, KT conference, KT social media channels, KT networks)
Item 7: Tailoring?	If the IKT strategy was planned to be personalised, then describe what, why, when, and how	To what extent was your IKT strategy tailored to the individual stakeholders
		Please describe how you personalised or tailored the IKT strategies to respective stakeholders. Feel free to provide examples!
		To what extent was your IKT strategy modified during the course of CEBHA+? i.e. Did you add or remove stakeholders from your strategy? Did you change the mode of engagement?
Item 8: Modifications	If the IKT strategy was modified during the course of the study, describe the changes (what, why, when, and how)	For what reasons were your IKT strategies modified? Please select all that apply.
		Please add other reasons why you had to modify your IKT strategies.
Item 9: How well	How well you planned and actually implemented your IKT strategy	Compared to how you originally planned your IKT strategy, to what extent did you adhere to this strategy?
